# CRISPR/Cas9-mediated *PD-L1* knockout reshapes temporal transcriptomic responses to PRRSV infection in porcine alveolar macrophages

**DOI:** 10.1371/journal.pone.0355142

**Published:** 2026-11-10

**Authors:** Sunirmal Sheet, Hwan-Ju Kim, Won-Il Kim, Seokho Kim, Poongyeon Lee, Ara Cho, Dahye Kim

**Affiliations:** 1 Animal Biotechnology and Genomics Division, National Institute of Animal Science, Rural Development Administration, Wanju, Republic of Korea; 2 College of Veterinary Medicine, Jeonbuk National University, Iksan, Republic of Korea; 3 Division of Animal Diseases & Health, National Institute of Animal Science, Rural Development Administration, Wanju, Republic of Korea; Cairo University Faculty of Veterinary Medicine, EGYPT

## Abstract

Porcine reproductive and respiratory syndrome virus (PRRSV) suppresses innate antiviral immunity in porcine alveolar macrophages, and the *PD-1*/*PD-L1* axis is thought to contribute to this immune dysregulation. This study investigated how *PD-L1* knockout (KO) alters the host transcriptional response to PRRSV infection compared to wild-type (WT) immortalized porcine alveolar macrophages (iPAMs). *PD-L1* KO iPAMs were generated by CRISPR/Cas9 targeting exon 2 of porcine *CD274* locus, and knockout was confirmed in clone #35. After PRRSV-2 infection, KO#35 cells exhibited approximately 5-fold reduced viral RNA accumulation at 48 h post-infection compared with WT cells. Time-course RNA sequencing at 0, 6, and 48 h post-infection was then performed to characterize the transcriptional consequences of *PD-L1* KO during acute PRRSV-2 infection. *PD-L1* KO iPAMs exhibited downregulation of immune checkpoint and exhaustion-related genes (*LAG3*, *PDCD1*, *TIGIT*, *TOX* family) and NF-κB inhibitors (*SOCS4*, *NFKBIA*), alongside upregulation of antigen presentation (MHC I/II genes like *SLA-2*, *SLA-5*, *B2M*, *SLA-DMA*, *SLA-DMB*, *HLA-DRA*) and co-stimulatory molecules (*CD80*, *CD86*, *CD40*). Cytokine profiling indicated a reprogrammed response favoring antiviral signaling, including sustained elevation of *CXCL10/11*, ISGs (*MX1/2*, *ISG15*, *IFIT1–3*), and select interleukins (*IL15*, *IL6*), while suppressing pro-inflammatory *TNF* and *CXCL8* across time points. Pathway enrichment analysis revealed significant enrichment of the cytokine–receptor interaction pathway. Weighted gene co-expression network analysis (WGCNA) further identified a key co-expression module containing 1,763 DEGs that was associated with innate immune activation. Moreover, protein-protein interaction (PPI) network analysis detected *PXDN* as a key hub gene connected to the PI3K-Akt signaling and natural killer cytotoxicity pathways. These findings provide transcriptomic insight into the association between *PD-L1* deficiency and altered macrophage transcriptional responses to PRRSV infection in an *in-vitro* iPAM model and provide a basis for future mechanistic investigation of *PD-L1* in PRRSV pathogenesis.

## Introduction

Porcine reproductive and respiratory syndrome virus (PRRSV) is a small, enveloped, single-stranded; positive-sense RNA virus classified in the genus *Betaarterivirus* (family *Arteriviridae*, order *Nidovirales*). It is one of the most economically important pathogens in the global swine industry, responsible for respiratory diseases in piglets and reproductive failure in sows due to its efficient transplacental transmission during late gestation. Infections occurring after approximately 72 days of gestation are strongly associated with high fetal mortality, resulting in abortions, premature farrowing, stillbirths, fetal mummification, and weak neonatal piglets [1]. Furthermore, PRRSV infection predisposes animals to secondary bacterial and viral infections, thereby exacerbating disease severity and increasing herd mortality [2].

A key feature of PRRSV pathogenesis is the virus’ ability to manipulate innate immunity in host cells, particularly porcine alveolar macrophages (PAMs), which are the primary target cells for viral replication in the lungs [3]. PAMs generally initiate early antiviral defenses by sensing pathogens and activating type I interferon pathways, resulting in further cytokine and chemokine production. However, PRRSV employs multiple viral antagonistic strategies to suppress these responses and delay and attenuate antiviral activity. Despite extensive vaccination and biosecurity efforts, sustainable control of PRRSV remains elusive, as current vaccines offer only partial protection because of the virus’s high genetic variability and complex interactions with the host immune system. Determining the molecular mechanisms underlying PRRSV-induced immune suppression is critical for identifying host targets to enhance antiviral resistance.

Programmed cell death ligand 1 (*PD-L1/CD274*) is a well-characterized immune checkpoint molecule that negatively regulates immune activation by engaging its receptor, PD-1. *PD-L1* is constitutively expressed or inducible across a diverse array of hematopoietic and non-hematopoietic lineages, spanning T and B lymphocytes, dendritic cells (DCs), macrophages, and endothelial cells [4,5]. *PD-L1* expression is often induced as a host regulatory mechanism to control excessive inflammation during viral infections [5]. However, sustained or excessive *PD-L1* activity promotes immune exhaustion and facilitates viral infection. Upregulation of *PD-L1* has been reported during infection with several viruses, including PRRSV [6], adenovirus [7], Ebola virus [8], hepatitis B virus (HBV) [9], lymphocytic choriomeningitis virus [10], respiratory syncytial virus [11], influenza virus [11], herpes simplex virus [12], human immunodeficiency virus [13], and rabies virus [14]. In the context of PRRSV infection, the functional role of *PD-L1* expression in PAMs and its impact on macrophage transcription and innate antiviral signaling remain largely unexplored.

Recently, CRISPR/Cas9 genome editing technology has emerged as a powerful and precise approach for studying host gene function by enabling targeted gene KO in relevant primary or immortalized cell types. CRISPR/Cas9-mediated *PD-L1* KO in PAMs offers a unique opportunity to directly assess how removal of this immune checkpoint reshapes host antiviral responses during PRRSV infection. Previous literature have successfully employed CRISPR/Cas9 to generate gene KOs conferring resistance to PRRSV infection, including deletions of *CD163*, its *SRCR5* domain, and the *PSTII* domain, highlighting the utility of this approach in identifying host factors critical for viral entry and replication [15]. Moreover, identification of candidate genes offers a valuable biological context for interpreting transcriptomic changes associated with different stages of viral infection in the host.

In this study, we knocked out *PD-L1* gene in an immortalized porcine alveolar macrophage (iPAM) model using CRISPR/Cas9 to investigate host immune responses to PRRSV infection. Following PRRSV-2 infection, viral replication kinetics were assessed via RT-qPCR in WT and *PD-L1* KO cells. We then performed comprehensive RNA sequencing (RNA-seq) at 0, 6, and 48 h post-infection in WT and KO cells. This time-course transcriptomic analysis allowed us to find phase-specific gene expression profiles changes across early and peak infection stages. Together, these findings offer a foundation for further investigation into the role of *PD-L1* in regulating host immune responses during PRRSV infection.

## Materials and methods

### Cell culture and virus strain

iPAM cells (iPAM‑KNU, hTERT; Abm) were cultured in Roswell Park Memorial Institute 1640 medium (RPMI-1640) (Gibco, Life Technologies, USA) containing L-glutamine, supplemented with 10% fetal bovine serum (FBS; Gibco), and 1% penicillin-streptomycin solution (Gibco) and maintained at 37 °C in a humidified 5% CO_2_ incubator. PRRSV-2 reference strain JBNU-23-N05 (GenBank accession no. PV459697) was used for infection experiments because of its representative clinical presentation and robust viremia levels, which facilitate the study of viral-host interactions in vitro [16]. Viral stocks were propagated in iPAMs cells. To facilitate CRISPR/Cas9-mediated knockout of the *PD-L1* gene, single guide RNA (sgRNA) was designed targeting the *Sus scrofa CD274* (*PD-L1*) gene sequence (RefSeq: NM_001025221.1). The online design tool CHOPCHOP (v3) was utilized, with the Sscrofa11.1 reference genome, selecting for high on-target efficiency and minimal off-target effects.

### CRISPR/Cas9-mediated *PD-L1* KO, clone selection, and confirmation

In the current study, *PD-L1* KO in iPAMs was performed using Lipofectamine 3000 (Thermo Fisher Scientific) according to the manufacturer’s specifications as detailed in previous reports [17]. Briefly, iPAM cells were plated at 1 × 10^5^ cells/well in 24-well plates and transfected at 70–80% confluence with a 1:1 mixture of U6-*PD-L1*-sgRNA plasmid and Cas9-Puro-GFP co-expression plasmid. Plasmid DNA (2.5 μg each) was mixed with P3000 reagent in 250 μL Opti-MEM, and Lipofectamine 3000 reagent was diluted separately in 50 μL Opti-MEM. After 5 min, the mixtures were pooled in one tube, incubated for 20 minutes at ambient temperature, subsequently introduced to the cell culture medium. After 6 h, the medium was switched with fresh RPMI‑1640 medium. GFP-positive cells were confirmed at 2 days post-transfection, and puromycin selection was performed over 5 days. Monoclonal cell populations were established via limiting dilution in 96-well plates to ensure the isolation of distinct single-cell clones, and used for further analysis. *PD-L1* sgRNAs and Cas9-Puro-GFP plasmids were synthesized by Bioneer Pharma (Seoul, Korea). Genome editing was initially screened by genomic cleavage detection assay. Further, reverse transcription-quantitative PCR (RT-qPCR) and western blot analysis were carried out for *PD-L1* KO confirmation. Subsequently, final candidate knockeout clone was validated by Sanger sequencing of PCR amplicons spanning the target site. Single-cell clone was subjected to direct Sanger sequencing by a commercial sequencing service provider Bionics, Korea.

### Initial genomic cleavage screening

Initial assessment of genome editing efficiency was performed using a genomic cleavage detection assay using Invitrogen GeneArt Genomic Cleavage Detection Kit according to the manufacturer’s protocol. Genomic DNA was isolated from transfected iPAM cells, and the region surrounding the CRISPR/Cas9 target site was amplified by PCR using specific primers (S1 Fig). The PCR products were then denatured and gradually re‑annealed to generate heteroduplexes between WT and edited alleles, followed by digestion with a mismatch‑sensitive nuclease supplied in the kit. Cleavage fragments were separated by agarose gel electrophoresis, and the appearance of lower‑molecular‑weight bands corresponding to nuclease‑dependent cleavage was interpreted as evidence of successful editing at the target locus.

### RT-qPCR analysis for *PD-L1* KO confirmation

Following the manufacturer’s instructions, TRIzol reagent 250 (Invitrogen, USA) was used to extract total RNA from iPAM cells. RNA quantity and purity were assessed spectrophotometrically at 260 nm and the 260/280 nm ratio using a SpectraMax Plus microplate reader (Molecular Devices). The Invitrogen SuperScript III First-Strand Synthesis System was used to reverse transcribe 1 μg of total RNA for cDNA synthesis, as directed by the manufacturer. Quantitative PCR analysis was carried out on the synthesized cDNA using the QuantStudio 3 Fast Real-Time PCR System (Applied Biosystems) with PowerUp™ SYBR™ Green Master Mix (Applied Biosystems™). Gene-specific primers for porcine (S1 Fig) were provided. Relative gene expression levels were calculated using the 2^−ΔΔCt^ method, normalizing the threshold cycle (Ct) values of target genes to the housekeeping gene *GAPDH*.

### Western blot analysis for *PD-L1* KO confirmation

To analyze PD-L1 KO at the protein level in the selected clone, western blot analysis was performed. Prior to protein extraction, recombinant interferon-gamma (IFN-γ; Abclonal, RP01070)) was stimulation was employed specifically prior to protein-level analysis because basal *PD-L1* protein expression in unstimulated iPAMs is characteristically low and insufficient for reliable detection by western blot. Therefore, recombinant porcine IFN-γ at a concentration of 100 ng/mL for 24 h in confluent iPAM cell monolayers, a dose consistent with previously published studies [18]. Western blot analysis was conducted as previously described [19,20]. Briefly, cells were harvested by scraping into RIPA buffer supplemented with 1% Halt protease and phosphatase inhibitor cocktail (Thermo Fisher, 78440) and kept on ice for 20 minutes. Subsequently, centrifugation at 14,000 rpm for 15 minutes under 4°C was followed and then carefully supernatants were collected. Bradford protein assay kit was used for Protein concentrations measurement (Pierce, 23227) as per company’s protocol. Equal amounts of protein (30 µg) were resolved on 10% SDS-PAGE gels (Bio-Rad, Cat. No. 161–1106) and transferred onto polyvinylidene fluoride (PVDF) membranes (Millipore, IPVH00010) using the Bio-Rad mini Trans-Blot cell system at 100 V for 2 h. Membranes were blocked for one hour at room temperature using 5% BSA in Tris-buffered saline with 0.1% Tween-20 (TBST), and they were then treated with *PD-L1* primary antibody (Invitrogen, PA5–119355) diluted in 5% BSA–TBST (typically 1:500 for polyclonal antibody) for an overnight at 4°C. Following three washes with TBST (10 min each); membranes were placed for 1 hour at room temperature with HRP-conjugated secondary antibody (anti-Rabbit; Cell Signaling Technology) at 1:2000 dilution in 5% BSA–TBST. Protein bands were spotted using Thermo Scientific Pierce ECL Western Blotting Substrate, and images were taken using a Bio-Rad GelDoc XR+ system.

### Virus titration, infection and measurement of PRRSV RNA abundance

Virus titration, infection, and quantification of PRRSV RNA abundance were performed as previously described in our study, with minor modifications [16,21–23]. PRRSV-2 strain JBNU-23-N05 was propagated in iPAMs maintained in RPMI-1640 medium supplemented with 10% FBS, and 1% penicillin-streptomycin and 0.25 µg/mL amphotericin B at 37°C in a humidified 5% CO_2_ incubator. After virus propagation, virus-containing cell lysates were clarified by centrifugation at 4,000 rpm for 30 min at 4°C, filtered through a 0.22-µm membrane filter, and stored as viral stocks [24]. Infectious virus titers were determined using a microtitration infectivity assay. Briefly, serial 10-fold dilutions of virus-containing samples were prepared and inoculated in quadruplicate onto confluent iPAM cell monolayers in 96-well plates. After 1 h of incubation at 37°C, the inoculum was removed, fresh RPMI growth medium was added, and cells were incubated for 5–6 days. Cytopathic effects were monitored daily, and viral titers were calculated based on CPE-positive wells. Virus titers were calculated based on CPE scores and expressed as the 50% tissue culture infective dose per milliliter (TCID₅₀/mL) according to the Reed–Muench method [23].

For PRRSV RNA abundance experiments, iPAMs were seeded in 6-well plates at a density of 1 × 10^5^ cells/well and allowed to adhere overnight at 37°C prior to infection. PRRSV-2 (JBNU-23-N05) was diluted in serum-free RPMI-1640 medium and added to cells at a multiplicity of infection (MOI) of 0.01, calculated based on the previously determined TCID₅₀/mL titer [21].

Following the 1 h adsorption period, the inoculum was removed, cells were washed twice with PBS to remove unbound virus, and fresh growth medium was added. Supernatant collected immediately thereafter was defined as the 0 h post-infection sample. Further, supernatants were collected at 6 and 48 h post‑infection for viral RNA. Viral RNA from collected supernatant samples was extracted using a viral nucleic acid extraction kit with a Tianlong automatic nucleic acid extractor (NP968, Xi’an Tianlong Science & Technology Co., China), according to the manufacturer’s instructions. RT-qPCR for quantifying PRRSV RNA abundance was conducted using a Prime-Q PCV2/PRRSV Detection Kit (GenetBio, Korea). To compare changes in PRRSV RNA abundance between cell types and time points, viral RNA levels were expressed using the − ΔCt method, whereby individual Ct values at each time point were subtracted from the mean Ct value of the corresponding 0 h post-infection baseline group for each experimental group. Fold changes in detectable viral RNA abundance relative to the 0 h post-infection baseline were calculated as 2^−ΔCt^.

### iPAM cell RNA isolation, sequencing, data processing and analysis

iPAMs were seeded in 6‑well plates at a density of 1 × 10^5^ cells per well in RPMI‑1640 medium and cultured overnight to approximately 80% confluence. Cells were then infected with PRRSV at a MOI of 0.01 and incubated for 48 h at 37 °C in a 5% CO_2_ atmosphere. For the 0 h post-infection condition, cells were exposed to PRRSV for 1 h to allow viral adsorption, after which the inoculum was removed and cells were washed with PBS and immediately harvested for RNA isolation. Similarly, cells were harvested at 6, and 48 h post-infection after a single wash with phosphate-buffered saline (PBS). Each experimental group was prepared with three independent biological replicates (n = 3), resulting in 18 RNA samples subjected to downstream sequencing and transcriptomic analysis. Total RNA was extracted from all samples using the TRIzol-chloroform-isopropanol fractionated method in accordance with the manufacturer’s instructions (Invitrogen, Carlsbad, CA, USA). RNA-quality assessment, next-generation sequencing (NGS) library preparation, sequencing, bioinformatics processing, quality control, read mapping, and differential expressions (DEs) analysis were implemented as mentioned before in our study [25]. Sequencing was conducted on an Illumina NovaSeq 6000 platform to generate paired-end reads. The RNA-seq libraries were sequenced using 150-bp paired-end sequencing with an unstranded (non-directional) library preparation protocol. Raw BCL files were converted to FASTQ format using bcl2fastq. Read quality assessment and trimming were performed using FastQC (v0.11) and Trimmomatic (v0.39) in paired-end mode by trimming ambiguous N bases and filtering reads with a Phred quality score < 20. Clean reads were aligned to *Sus scrofa* reference genome Sscrofa11.1 (version 114) using HISAT2 (v2.2.1). Gene-level counts were quantified from aligned BAM files using featureCounts (Subread package v2.0.1), and the resulting count matrix was used for downstream analyses. The read quality, mapping performance, gene-assignment rates, and sample-level quality control results are provided in S1 Table.

### Data normalization and differentially expressed gene (DEG) analysis

The resulting raw integer count matrix from featureCounts was supplied directly to DESeq2 (v1.42.1) package in R (R v4.3.3) without prior normalization or transformation. Lowly expressed genes with a total count of <10 across samples were filtered out, after which DESeq2 size-factor normalization and differential-expression analysis were performed. Differential expression was evaluated using separate pairwise comparisons, with an independent DESeq2 model fitted for each comparison using the design formula ~ condition, where condition was treated as a categorical two-level factor representing the two groups being compared. For genotype comparisons at each time point, WT was used as the reference group, and the contrasts were specified as WT_0 vs. KO_0, WT_6 vs. KO_6, and WT_48 vs. KO_48. Temporal changes within each genotype were also assessed through separate pairwise comparisons among the 0, 6, and 48 h time points (e.g., KO_0 vs. KO_6, KO_0 vs. KO_48, and KO_48 vs. KO_48). Thus, each time point was analyzed as a distinct categorical condition, and time was not modeled as a continuous variable. No overall likelihood-ratio test or genotype-by-time interaction test was performed. For visualization, count data were transformed using the regularized logarithm (rlog) transformation with blind = FALSE after construction of the DESeq2 dataset. A padj  <  0.05 (adjusted p-value) and log2 fold-change (FC)  ≥  1.5 were set as the cut-off criteria for screening DEGs. The transformed values were used for PCA and heatmap visualization and were not used for differential-expression statistical testing. The obtained DEGs were classified into up-regulated and down-regulated genes following the log2FC value. The PCA plot, volcano plot, and heatmap were explored and visualized using ggplot2 (v4.0.1) and pheatmap (v1.0.12) in R language, respectively. The overlapped DEGs between different datasets were identified using interactive E Venn, a web-based tool.

### Functional enrichment analysis

To characterize signaling pathways and biological functions associated with PRRSV infection, DEGs were subjected to Gene Ontology (GO) and Kyoto Encyclopedia of Genes and Genomes (KEGG) enrichment analyses using the Database for Annotation, Visualization, and Integrated Discovery (DAVID; https://david.ncifcrf.gov/) and clusterProfiler R package (v4.17.0) [25,26]. *Sus scrofa* was specified as the background/reference genome, and statistical significance was determined using a threshold of adjusted p-value < 0.05.

### Weighted gene co-expression network analysis (WGCNA) and key modules identification

WGCNA was performed as an exploratory analysis using all 18 samples from the six experimental conditions, as previously described [26]. Raw integer gene-level counts were used to construct a DESeq2 dataset with the design formula ~ condition, and genes with a total count <10 across samples were excluded. Variance-stabilizing transformation (VST) was subsequently performed using vst (blind = TRUE). Thus, the transformation was performed independently of the experimental design and was used solely to stabilize the mean–variance relationship of the count data. It was not used for batch-effect removal. No batch correction was applied prior to WGCNA, as all 18 RNA-seq libraries were generated from the same iPAM cell line in a single experimental batch under controlled in vitro conditions with no identified technical confounders; accordingly, the full biological signal reflecting genotype (WT vs KO) and infection time point was preserved in the transformed expression matrix.

Six predefined pairwise differential-expression analyses were performed, and genes meeting the criteria of padj ≤ 0.05 and log2FC ≥ 1 in at least one comparison were combined to generate a DEG union. The VST-transformed expression matrix was then restricted to this DEG union and used as input for WGCNA. Sample quality was assessed by hierarchical clustering and the goodSamplesGenes function before network construction. A signed co-expression network was constructed using the WGCNA package (v1.73). Scale-free topology was evaluated across candidate soft-thresholding powers from 1 to 30 using pickSoftThreshold, with a target scale-free topology fit of R^2^ ≥ 0.80. A soft-thresholding power of 18 was selected based on the scale-free topology criterion while retaining adequate mean connectivity. The adjacency matrix was calculated under a signed network assumption and transformed into a signed topological overlap matrix (TOM). TOM-based dissimilarity (1 − TOM) was subsequently used for average-linkage hierarchical clustering of genes.

Gene modules were identified using the dynamic tree cut algorithm (cutreeDynamic) with deepSplit = 2 and a minimum module size of 30 genes. Closely related modules were merged based on module eigengene similarity using a merge cut height of 0.30. Module eigengenes (MEs) were calculated for the resulting modules and correlated with the predefined experimental contrast indicators using Pearson correlation with pairwise-complete observations. Corresponding nominal p-values were estimated using the Student asymptotic test and adjusted for multiple comparisons across the complete set of module-trait tests using the Benjamini-Hochberg procedure. Module-trait relationships were visualized using a module–trait correlation heatmap displaying the correlation coefficients and FDR-adjusted p-values. Because of the relatively small sample size, the identified WGCNA modules were interpreted as exploratory co-expression patterns rather than independently validated or reproducible network structures.

### Protein–protein interaction (PPI) network construction and module identification

A PPI network was constructed by uploading the 1763 DEGs identified from the top WGCNA blue module to the STRING database. The resulting interaction data were imported into Cytoscape (v3.10.3) for network visualization and downstream analysis. To identify densely connected subnetworks (hub clusters), the MCODE plugin (v2.0.3) was applied, and top-ranked modules were extracted using the following parameters: node score cutoff = 0.2, degree cutoff = 2, maximum depth = 100, and k-core = 2.

### Statistical analysis

Statistical analyses were performed in Prism (GraphPad ver. 8.4, San Diego, U.S.A). Virus replication fold-changes were compared using two-way ANOVA followed by a post-hoc test. Statistical significance was set as *p < 0.05, ** p < 0.01.

## Results

### Confirmation of *PD-L1* KO in iPAM cells and viral gene expression

The effect of *PD-L1* was assessed using CRISPR/Cas9 gene editing to generate a *PD-L1* KO single-cell clone in iPAM cells and by confirming gene deletion (Fig 1). To establish the *PD-L1* knockout iPAM cell lines, a sgRNA was designed to target exon 2 region of the porcine *PD-L1* transcript NM_001025221.1, adjacent to a canonical AGG protospacer-adjacent motif (PAM) at genomic position NC_010443.5:216675206. Two separate expression vectors were constructed, in which Cas9 and Puro-RFP were placed under the control of the CMV/T7 promoter, whereas the *PD-L1*-targeting sgRNA was driven by the U6 promoter (Fig 1B). After plasmid transfection into iPAM cells, the successfully transfected cells were enriched by puromycin selection and fluorescence-based screening, and single-cell clones were isolated and expanded for further characterization. Initial screening of transfected cells using a cleavage assay identified multiple candidate edited clones #5, # 33, #35, and #37 (S1 Fig B). In addition, RT-qPCR analysis was performed for four selected clones and found that *PD-L1* expression was not detected in KO clone #35 compared with that in WT cells (Fig 1C). Furthermore, we have carried out protein level confirmation for KO clone #35 under both basal and IFN-γ-stimulated conditions. Our result showed that the *PD-L1* protein was detectable in WT cells, whereas no detectable protein band was observed in KO cells (Fig 1D). Subsequent Sanger sequencing of the targeted locus confirmed the presence of an 8-bp deletion in the selected KO clone #35 (Fig 1E). The *PD-L1* KO#35 cell line was used for subsequent experiments.

**Fig 1:**
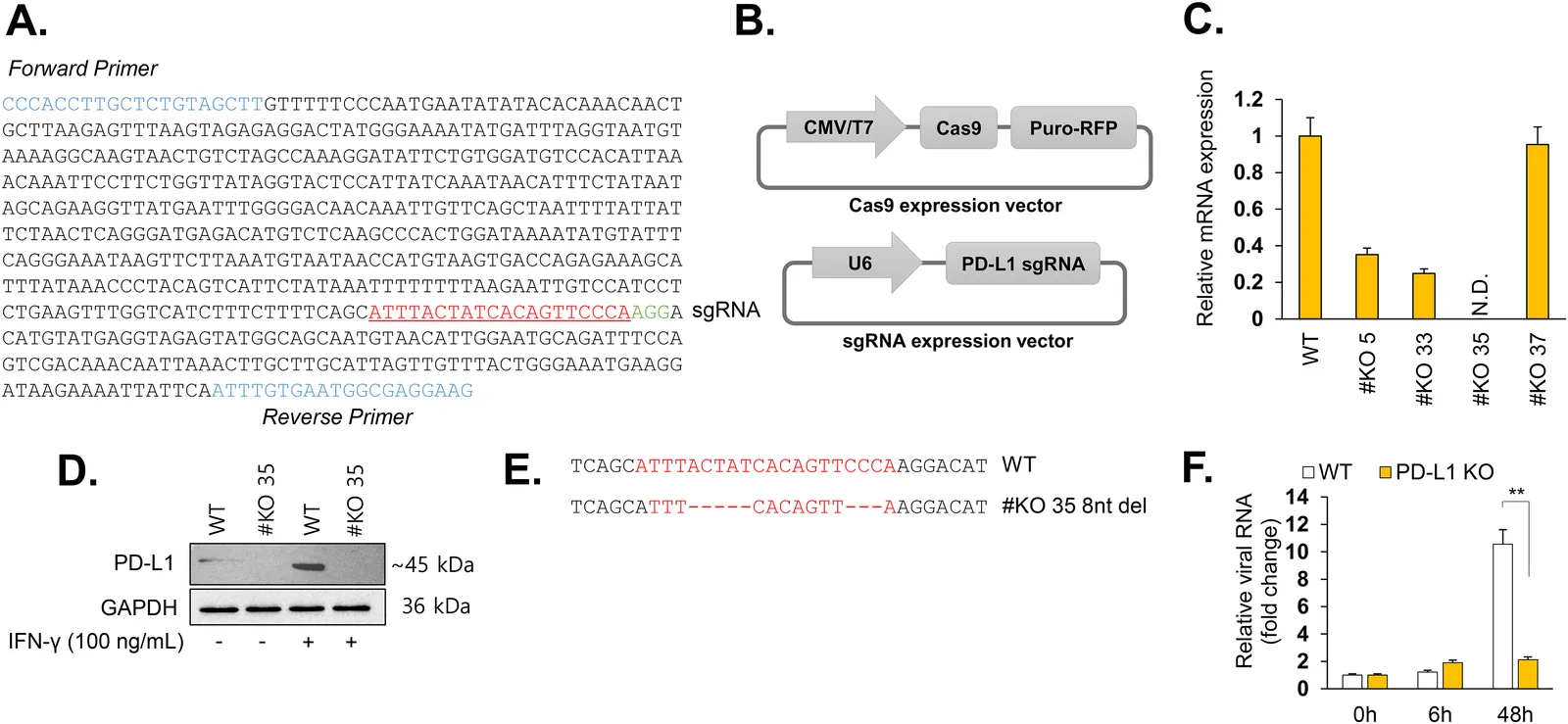
Generation of *PD-L1* gene KO in iPAM cell. (**A**) Schematic representation of the genomic target region in the porcine *PD-L1* locus (NM_001025221.1), showing the positions of the forward and reverse genomic PCR primers, and sgRNA target sequence. (**B**) Diagram of the Cas9 expression vector and the sgRNA expression vector. (**C**) Relative *PD-L1* mRNA expression in WT and KO clones. (**D**) Western blot analysis of *PD-L1* expression in WT and KO cells at both basal and IFN-γ-stimulated conditions. (**E**) Direct Sanger sequencing of the edited single-cell-derived KO clone #35. (**F**) Relative PRRSV RNA abundance, determined by RT-qPCR, in WT and KO cells at 0, 6, and 48 h post-infection. WT: Wild type; KO: Knockout; N.D.: Not detected.

Furthermore, PRRS viral RNA level in the *PD-L1* KO#35 cell line was measured at 0, 6, and 48 h post-infection and expressed as relative fold change normalized to the 0 h baseline (Fig 1F). The result confirmed a reduced virus RNA at 48 h post PRRSV infection. By 48 h post-infection, WT cells exhibited a marked increase in viral RNA, reaching an approximately 10.5-fold change relative to baseline. In contrast, *PD-L1* KO cells showed only a modest 2.1-fold increase at the same time point, representing an approximately 5-fold reduction in viral RNA accumulation compared to WT cells (P < 0.01).

### Differential gene expression profiles in *PD-L1* KO and WT iPAMs following PRRSV infection

To obtain insights into transcriptional alterations associated with PRRSV infection and *PD-L1* KO, we performed a high-throughput transcriptome analysis of total RNA isolated from *PD-L1* KO and WT iPAMs collected at 0, 6, and 48 h post‑infection. This time-resolved transcriptome profiling enabled us to characterize the dynamic changes in host gene expression in response to viral challenge and to compare infection-induced signatures between *PD-L1* KO and WT cells. The DEGs identified were subsequently used for downstream analyses to elucidate the pathways and biological processes underlying the altered antiviral and immune responses in *PD-L1* KO iPAMs. Principal component analysis (PCA) of the transcriptomic profiles demonstrated that PC1 (51% variance) and PC2 (28% variance) together accounted for 79% of the total variance, providing high-confidence separation of samples by both genotype and infection time point (Fig 2A). At early time points, WT_0 and WT_6 samples co-clustered tightly in the lower-central region of the PCA plot, while KO_0 and KO_6 samples co-clustered in the left region, indicating that early PRRSV infection (0–6 h post-infection) may induced relatively modest transcriptional divergence within each genotype. By contrast, both WT_48 and KO_48 samples were clearly separated from their respective early time-points, showing possible major transcriptional changes at 48 h post-infection in both genotypes. Next, we performed pairwise comparisons of DEGs across all six contrasts and summarized the similarity of DEG profiles using hierarchical clustering of the correlation matrix. The clustered correlation heatmap revealed two clearly separated modules (Fig 2B). Genotype contrasts at matched time points (WT_0 vs. KO_0, WT_6 vs. KO_6, WT_48 vs. KO_48) were characterized by strong positive correlations. The second cluster comprised *PD-L1* KO samples (KO_0 vs. KO_6, KO_0 vs. KO_48, KO_6 vs. KO_48), which showed comparatively weaker correlations.

**Fig 2:**
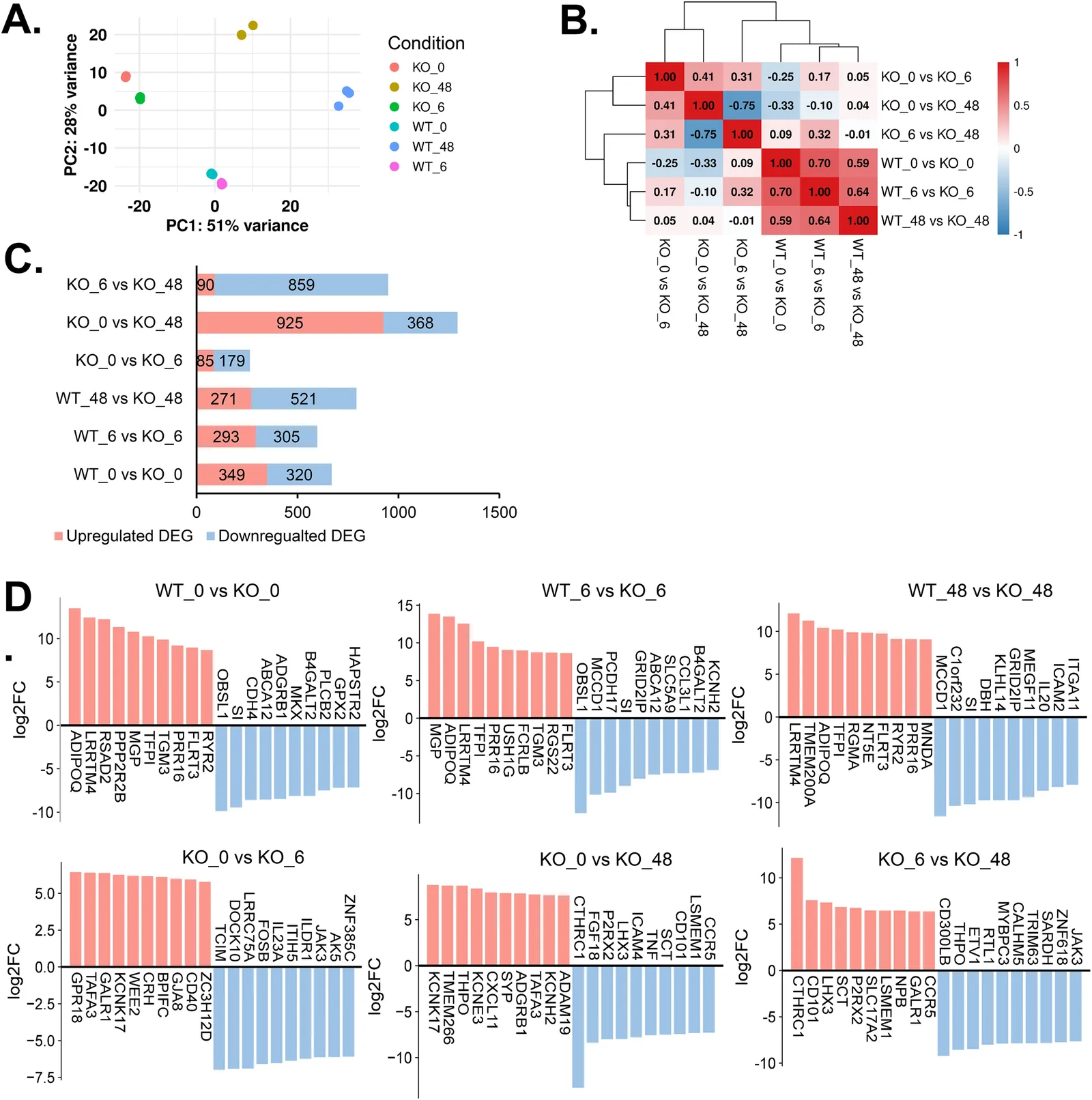
Transcriptomic profiling in WT and *PD-L1* KO iPAM cells after PRRSV infection. (**A**) Principal-component analysis showed clear clustering for each time points in WT and KO iPAM. PC1 and PC2 explained 51% and 28% of the total variance. (**B**) Correlation heatmap with hierarchical clustering showing the relationships among the six differential expression comparisons. The color scale represents the correlation coefficient. (**C**) Total numbers of significant upregulated and downregulated DEGs identified at different time points in WT and KO cells with padj≤0.05 and log_2_ fold-change ≥1.5. (**D**) Top 10 upregulated and top 10-downregulated genes in each comparison, ranked by log_2_ fold-change. Orange bars designate upregulated genes, and blue bars specify downregulated genes. WT: Wild type; KO: Knockout.

A padj  <  0.05 (adjusted p-value) and log2 fold-change (FC)  ≥  1.5 were set as the cut-off criteria for screening DEGs (Fig 2C and S2 Table). From the DEG analysis, a comparison of WT_0 vs. KO_0 identified 349 upregulated and 320 downregulated genes, WT_6 vs. KO_6 yielded 293 upregulated and 305 downregulated genes, and WT_48 vs. KO_48 showed 271 upregulated and 521 downregulated genes. Among the *PD-L1* KO iPAMs, KO_0 vs. KO_6 resulted in 85 upregulated and 179 downregulated genes, whereas KO_0 vs. KO_48 produced 925 upregulated and 368 downregulated genes, and KO_6 vs. KO_48 yielded 90 upregulated and 859 downregulated genes. Here, we highlight the top 10 significantly upregulated and downregulated genes for each pairwise comparison (Fig 2D). At 0 h, the anti‑inflammatory adipokine gene *ADIPOQ*, which encodes adiponectin, was the most strongly upregulated transcript (13.49‑fold) in WT_0 vs. KO_0. In the 6 h comparison (WT_6 vs. KO_6), matrix Gla protein (MGP), a regulator of extracellular matrix calcification and vascular homeostasis, showed the highest upregulation, whereas at 48 h (WT_48 vs. KO_48), *LRRTM4*, leucine rich repeat transmembrane neuronal 4, was the most highly upregulated (12.11-fold) gene. Within the *PD-L1* KO comparison, *GPR18*, G protein‑coupled receptor 18, was the leading upregulated (6.43-fold) gene in KO_0 vs KO_6; *KCNK17*, a two‑pore domain potassium channel associated with membrane potential and cellular excitability, was most upregulated (8.79-fold) in KO_0 vs KO_48; and *CTHRC1*, collagen triple helix repeat containing 1, was the top upregulated (12.13-fold) gene in KO_6 vs KO_48.

Heatmaps and volcano plots were generated for each time point and experimental group using normalized gene expression counts to visualize DEGs between samples (S2 Fig). The overlapping upregulated and downregulated DEGs identified in *PD-L1* KO cells across the three infection time points are illustrated in the Venn diagram shown in Fig 3. The corresponding lists of genes common to each group are provided in S3 Table. Among the upregulated DEGs identified from the three WT vs KO pairwise comparisons (WT_0 vs KO_0, WT_6 vs KO_6, and WT_48 vs KO_48), Venn diagram analysis revealed that 125 genes were commonly upregulated and 9 genes commonly downregulated across all three time-point comparisons (Fig 3). Within *PD-L1* KO cells (KO_0 vs KO_6, KO_0 vs KO_48, and KO_6 vs KO_48), temporal comparison of DEGs across infection time points identified 148 genes commonly upregulated and 3 genes commonly downregulated across all three KO internal comparisons (Fig 3). Moreover, the six-way star Venn diagram revealed only 6 upregulated genes and 0 downregulated genes shared across all six pairwise (WT_0 vs KO_0, WT_6 vs KO_6, WT_48 vs KO_48, KO_0 vs KO_6, KO_0 vs KO_48, and KO_6 vs KO_48) comparisons (Fig 3).

**Fig 3:**
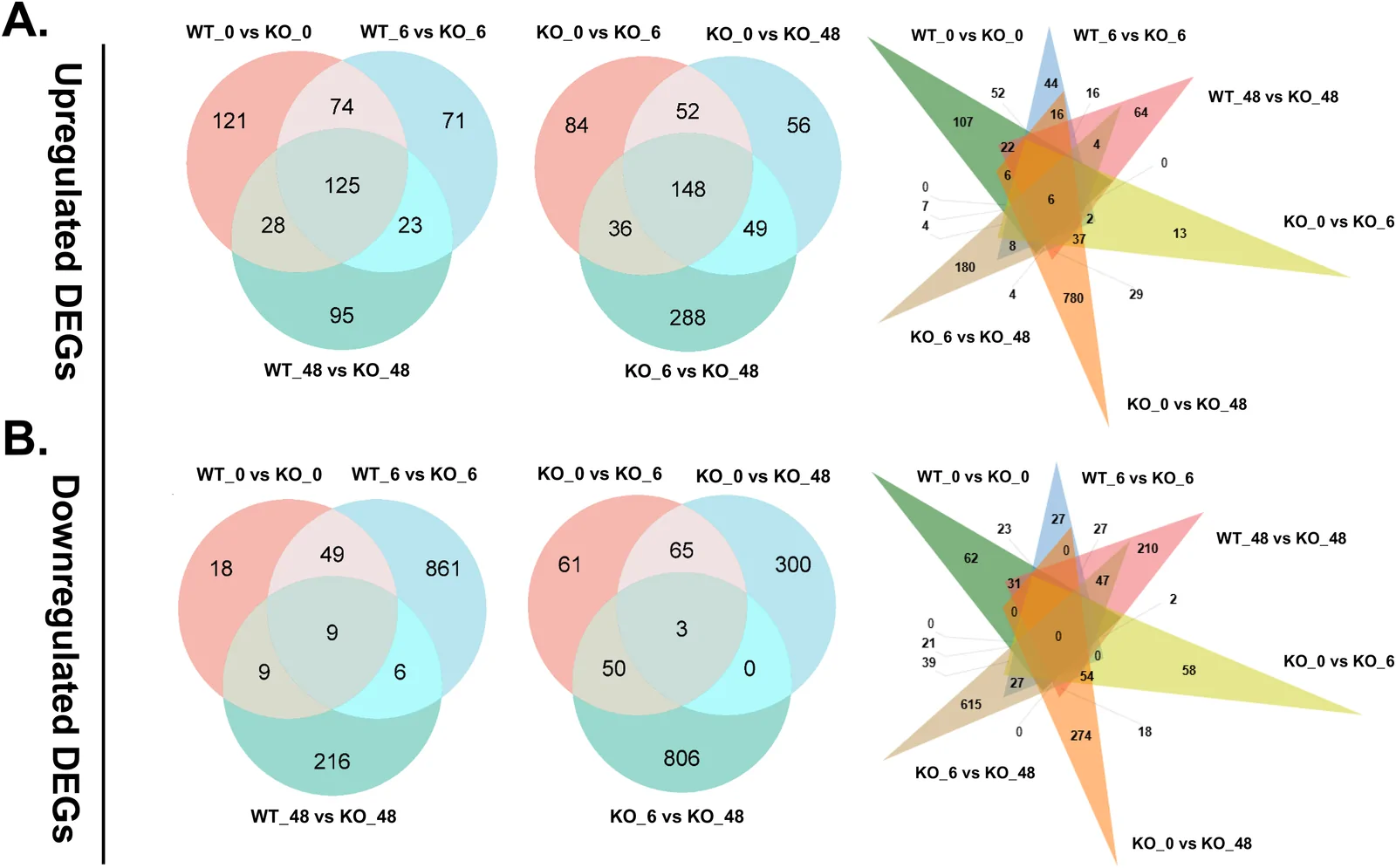
Venn diagrams showing overlapping DEGs of pairwise comparisons among three time points 0, 6, and 48 h of post infection in WT and *PD-L1* KO iPAM cells. (**A**) Upregulated DEGs. (**B**) Downregulated DEGs. WT: Wild type; KO: Knockout.

### Expression profile of immune regulatory genes during PRRSV infection in *PD-L1* KO cells

Based on recent studies, we systematically examined and prioritized DEGs involved in PRRSV infection and host defense immunity, including those linked to immune regulation especially immune checkpoint and exhaustion, NF‑κB signaling, and MHC class I and II antigen presentation and co-stimulatory regulation (Fig 4, S4 Table). Our differential expression analysis of 32 immune checkpoint and exhaustion-related genes revealed widespread suppression in *PD-L1* KO iPAMs compared to WT iPAMs at 0, 6, and 48 h post-infection (Fig 4A). Canonical exhaustion/checkpoint markers, including *LAG3*, *PDCD1*, *TIGIT*, and *PDCD1LG2* (*PD-L2*), were consistently downregulated throughout the course of infection, indicating broad attenuation of inhibitory signaling modules. In addition, exhaustion-controlling transcription factors such as *TOX*, *TOX2*, and *NR4A* exhibited reduced expression following *PD-L1* KO. However, compensatory immunosuppressive mechanisms emerged, with the potent tryptophan-catabolizing enzyme *IDO1* strongly upregulated (6.12-fold at 0 h, 4.91-fold at 6 h, 4.27-fold at 48 h). Expression of secondary checkpoint-associated gene *HAVCR2* (*TIM-3*) was upregulated at all-time points in *PD-L1* KO cells (3.11-fold at 0 h, 1.74-fold at 6 h, 1.88-fold at 48 h). Furthermore, *CD80*, a dual-function ligand capable of delivering both costimulatory and inhibitory signals, was markedly upregulated in *PD-L1*KO cells at all three time points.

**Fig 4:**
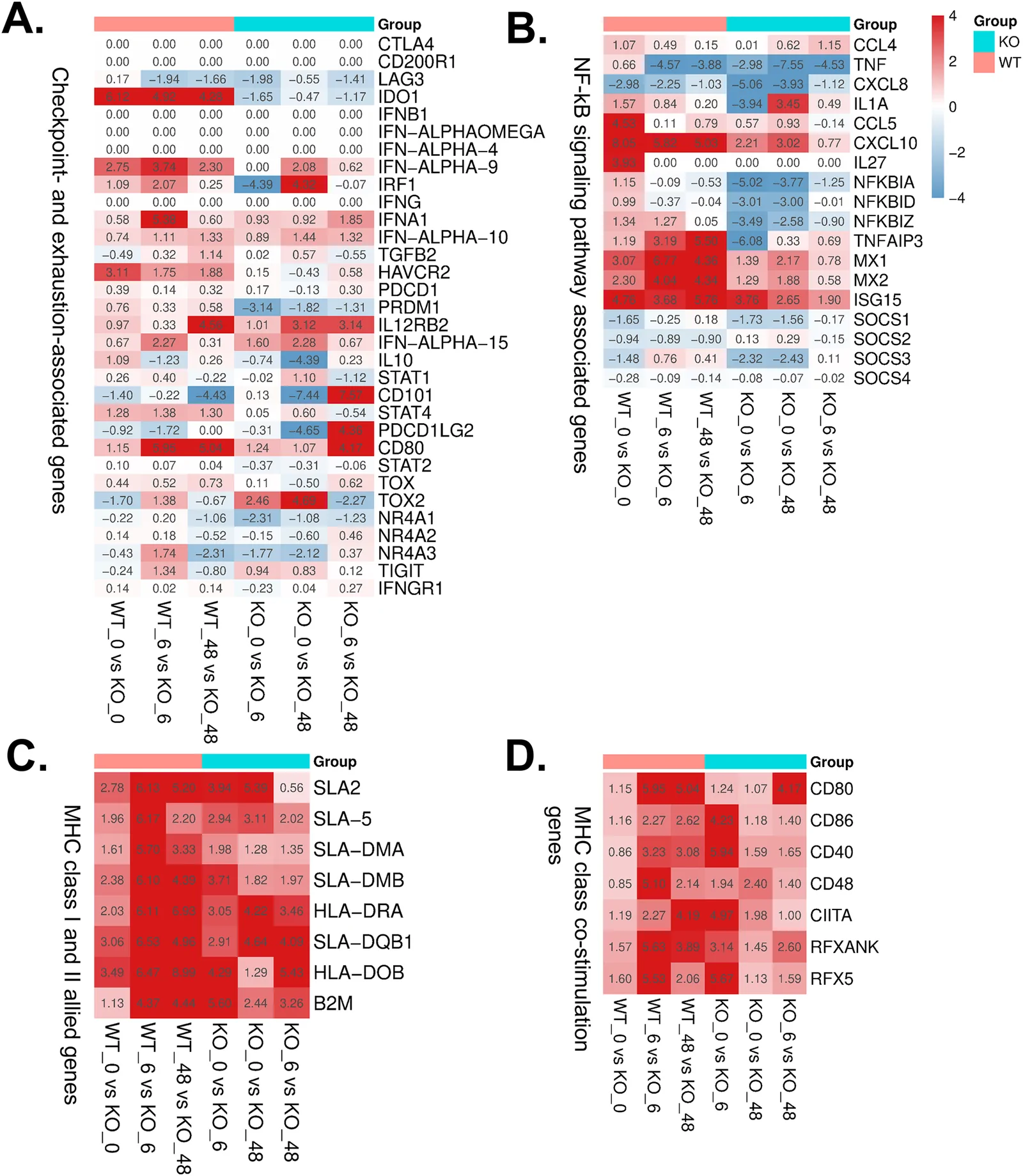
Heatmap plots of expression patterns of selected immune-related genes in WT and *PD-L1* KO PRRSV-infected cells across six pairwise comparisons (WT_0 vs. KO_0, WT_6 vs. KO_6, WT_48 vs. KO_48, KO_0 vs. KO_6, KO_0 vs. KO_48, and KO_6 vs. KO_48). (**A**) Checkpoint- and exhaustion-associated genes. (**B**) NF-kB signaling pathway allied genes. (**C**) MHC class I and II allied genes. (**D**) MHC class co-stimulation genes. WT: Wild type; KO: Knockout.

Given the relevance of inflammatory signaling during PRRSV infection, we curated 22 DEGs annotated as NF-κB pathway-regulated inflammatory genes (Fig 4B). In this study, *PD-L1* KO iPAMs showed robust induction of interferon/ISG-associated genes, including *CXCL10*, *MX1*, *MX2*, and *ISG15*, across all time points compared to WT cells, indicating markedly enhanced antiviral transcriptional activity. In contrast, canonical pro-inflammatory cytokines, including *TNF* and *CXCL8*, were downregulated in *PD-L1* KO cells across infection time points. Moreover, the expression of negative regulators of NF-κB activation such as *NFKBIA*, *NFKBID*, *NFKBIZ*, and *TNFAIP3* was consistently downregulated in *PD-L1* KO cells, which suggests prolonged NF-κB activity and sustained inflammatory signaling during PRRSV infection. Similarly, multiple suppressor of cytokine signaling (SOCS) family members (*SOCS1*, *SOCS2*, *SOCS3*, *SOCS4*), which are key negative feedback inhibitors of cytokine signaling, exhibited reduced expression in KO cells at most time points.

Subsequently, we examined expression changes in eight MHC class I/II pathway-associated DEGs, because antigen presentation capacity strongly influences downstream immune activation during viral infection (Fig 4C). Time-course comparisons in *PD-L1* KO cells at 0, 6, and 48 h post-infection revealed marked upregulation of multiple MHC-related DEGs. MHC class I pathway associated genes, including *SLA-2*, *SLA-5*, and *B2M*, alongside MHC class II loading/editing complex components (*SLA-DMA*, *SLA-DMB*), and α/β chain transcripts (*HLA-DRA*, *SLA-DQB1*, *HLA-DOB*), exhibited consistently elevated expression in *PD-L1* KO iPAMs. At 6 h post-infection, expression of all genes was greater than 4-fold in the WT vs. *PD-L1* KO comparison group. To link antigen presentation to functional activation potential, we evaluated seven DEGs encoding costimulatory ligands and transcriptional regulators of MHC expression (Fig 4D). Key activation/co-stimulation markers (*CD80*, *CD86*, *CD40*, *CD48*) and MHC transcriptional regulators (*CIITA*, *RFXANK*, *RFX5*) were significantly upregulated in *PD-L1* KO iPAMs.

### Cytokines expression profiles in PRRSV-infected *PD-L1* KO iPAMs

To examine how *PD-L1* KO alters inflammatory signaling during PRRSV infection, we analyzed curated cytokine families, including chemokines, interleukins (ILs), type I interferons (IFNs), TNF superfamily members, and their receptors across WT vs. *PD-L1* KO comparisons at 0, 6, and 48 h, and within *PD-L1* KO time-course changes (S4 Table). Dot plots and gene expression fold changes revealed the strongest transcriptional variation post-infection (Fig 5).

**Fig 5:**
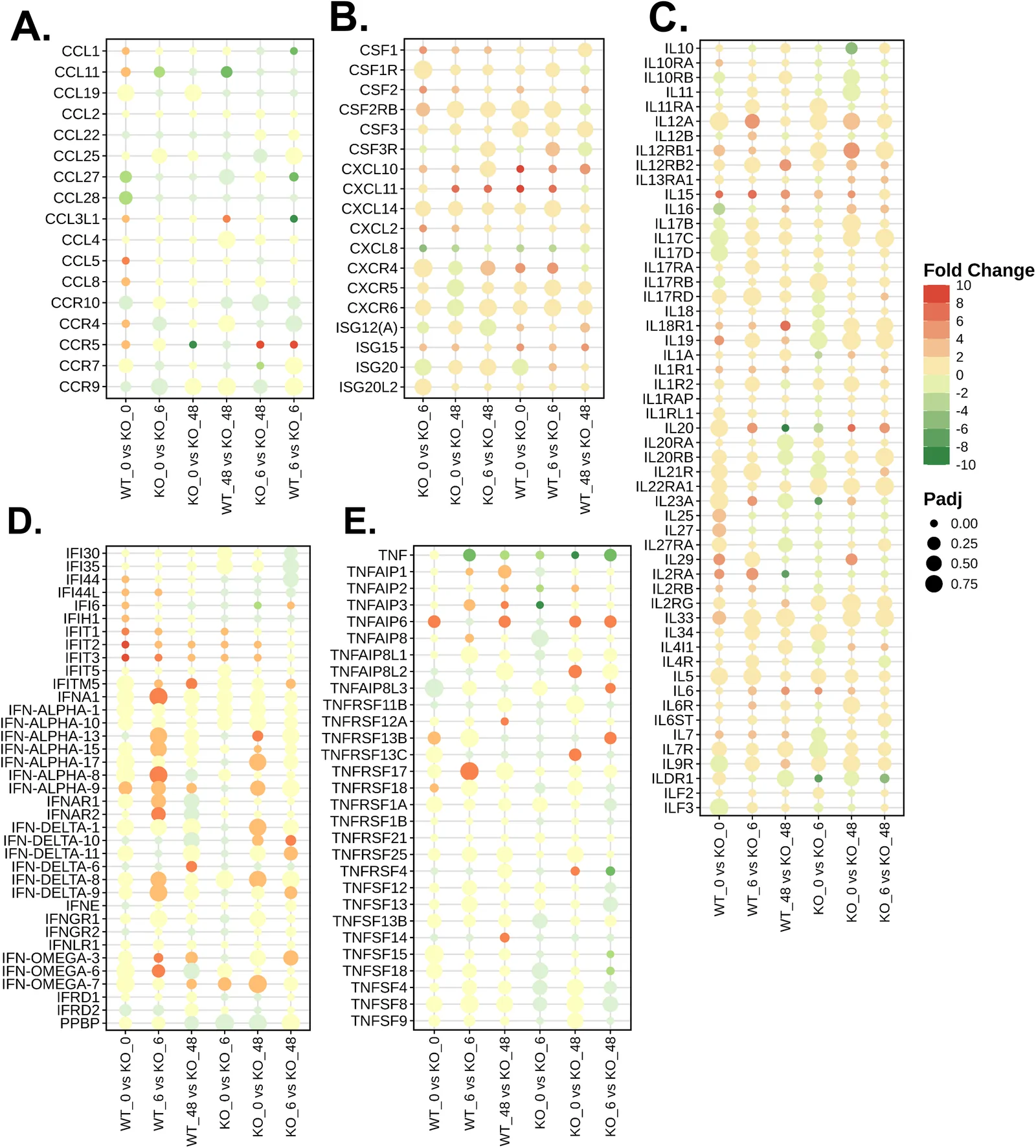
Gene expression patterns of cytokines and their receptors in six pairwise comparisons (WT_0 vs. KO_0, WT_6 vs. KO_6, WT_48 vs. KO_48, KO_0 vs. KO_6, KO_0 vs. KO_48, and KO_6 vs. KO_48) of WT and *PD-L1* KO PRRSV infected iPAM cells at three time points. (**A**) CCLs and their receptors. (**B**) CXCLs and related genes. (**C**) Allied genes of the ILs. (**D**) IFN related genes. (**E**) TNF- superfamily associated genes. WT: Wild type; KO: Knockout.

At 0 h post-infection, *PD-L1* KO iPAMs exhibited elevated baseline expression of several chemokines, including *CCL1* (3.16-fold), *CCL2* (1.92-fold), *CCL5* (4.53-fold), and *CCL8* (2.17-fold), along with increased expression of their receptors *CCR4* (2.12-fold) and *CCR5* (2.23-fold). In contrast, the levels of tissue-associated chemokines *CCL27* and *CCL28* were reduced, indicating that *PD-L1* deletion establishes a distinct basal chemokine landscape. At 6 h post-infection, chemokine expression pattern shifted markedly, with substantial downregulation of *CCL1* (−4.73-fold) and *CCL3L1* (−7.29-fold) relative to that in WT cells, coupled with robust upregulation of *CCR5* (6.40-fold), suggesting reprogramming of acute-phase chemokine–receptor signaling with *PD-L1* deficiency. After 48 h, expression divergence persisted, with continued elevation of *CCL3L1* (5.08-fold) and reduction in *CCL11* (−4.60-fold) in *PD-L1* KO cells. Notably, antiviral CX chemokines *CXCL10* and *CXCL11* remained highly upregulated (> 5-fold at 0 and 48 h) along with *CXCR4* (> 5-fold at 0 and 6 h) and *CSF2* across all time points, accompanied by strong induction of ISG15 (> 3-fold). In contrast, the neutrophil chemoattractants *CXCL8* (*IL-8*) and *CSF1R* were markedly downregulated. Sustained upregulation of *CXCL10/11*, *ISG15*, and *CSF2* from 0 to 48 h indicated a stable antiviral signaling phenotype in *PD-L1* KO iPAMs.

Among antiviral IFNs and their effectors, *IFIT1* (5.20-fold), *IFIT2* (6.51-fold), and *IFIT3* (7.56-fold) were strongly upregulated at 0 h and remained elevated at 6 and 48 h. Other IFN-stimulated genes (*IFI44*, *IFI44L*, *IFI6*, *IFIH1*) were consistently upregulated (> 2-fold) at all time points compared to those in WT cells. Multiple IFN ligands, including *IFNA1*, *IFNA1*, *IFN-ALPHA-8*, *IFN-ALPHA-9*, *IFN-ALPHA-13*, *IFN-OMEGA-3*, *IFN-OMEGA-6*, *IFN-DELTA-6*, *IFN-DELTA-8*, *IFN-DELTA-9*, and *IFN-DELTA-9* were predominantly upregulated at 6 h post-infection. At 48 h, only *IFN-DELTA-6*, *IFITM5*, and *IFIT3* were strongly expressed (> 3-fold increase). Within the *PD-L1* KO infection time course, *IFIT1*, *IFIT2*, and *IFIT3* were upregulated at 6 h, whereas *IFN-ALPHA-13*, *IFN-DELTA-8*, and *IFN-DELTA-10* were upregulated at 48 h. In contrast, the lysosomal IFN-associated regulators *IFI30* and *IFI35* were consistently downregulated across all time points.

We focused on the expression dynamics of ILs and members of the TNF superfamily during PRRSV infection. *PD-L1* KO iPAMs demonstrated strong upregulation (> 3-fold) of *IL15*, *IL2RA*, *IL19*, *IL29*, *IL33*, *IL25*, *IL27*, and *IL7* at 0 h. At 6 h post-infection, *IL15*, *IL20*, *IL23A*, *IL12A*, *IL2RA*, *IL6R*, *IL6*, and *IL18R1* were elevated (> 3-fold), whereas at 48 h, *IL18R1*, *IL6*, *IL15*, *IL12RB2*, and *IL7* remained upregulated relative to these genes in WT. Interestingly, several key pro-inflammatory and immunosuppressive cytokines such as *IL20*, *TNF*, *IL2RA*, and *IL10*, were notably downregulated (> 4-fold) in the *PD-L1* KO line, with TNF showing consistent suppression at all time points. Conversely, negative regulators of TNF/NF-κB signaling, including *TNFAIP3* (5.50-fold at 48 h) and *TNFAIP6* (4.69-fold at 48 h, 4.39-fold at 0 h), as well as members of the TNF receptor superfamily (*TNFRSF4*, *TNFRSF13B*, *TNFRSF13C*, *TNFRSF17*), were significantly upregulated following infection in *PD-L1* KO cells.

### Functional analysis KEGG pathway and GO enrichment analysis

For functional enrichment analysis, commonly dysregulated DEGs were extracted from the overlapping regions of each Venn diagram group: 125 upregulated and 9 downregulated DEGs shared across all three WT vs. KO comparisons, 148 upregulated and 3 downregulated DEGs shared across all three KO internal temporal comparisons, and 6 upregulated and 0 downregulated DEGs shared across all six pairwise comparisons simultaneously. Pooling these intersection-derived gene sets yielded a total of 291 DEGs, which were subsequently subjected to KEGG pathway and Gene Ontology (GO) enrichment analysis to elucidate their biological roles and pathway enrichment in *PD-L1* KO iPAMs during PRRSV infection. Using a significance threshold of p < 0.05, we identified 217 enriched Gene Ontology (GO) terms for biological processes (BP), 27 for molecular functions (MF), 10 for cellular components (CC), and 45 terms significantly associated with KEGG pathways. A complete list of these enriched GO terms and pathways is provided in S5 Table. The top significant (p < 0.05) immune-related pathways were cytokine-cytokine receptor interaction, chemokine signaling, TNF signaling, antigen processing and presentation, intestinal immune network for IgA production, Th17 cell differentiation, viral myocarditis, viral protein interaction with cytokine and cytokine receptor, IL-17 signaling, NF-kappa B signaling, and Toll-like receptor signaling pathways (Fig 6A). Among these, cytokine-cytokine receptor interaction was the top pathway enriched in the DEGs (Fig 6B). Expression of the upregulated and downregulated DEGs included in this pathway is shown in a heatmap (Fig 6B). In this pathway, important upregulated cytokines *CXCL10*, *CXCL11*, *IL6*, and *IL15* (> 3-fold) were enriched. Next, among the significant GO-BP categories, inflammatory response, GO-MF category, immune receptor activity, GO-CC category, and external side of plasma membrane were the top GOs to be enriched. The top 10 significant GO terms and their enriched DEGs expression profiles are shown in Fig 6C–E.

**Fig 6:**
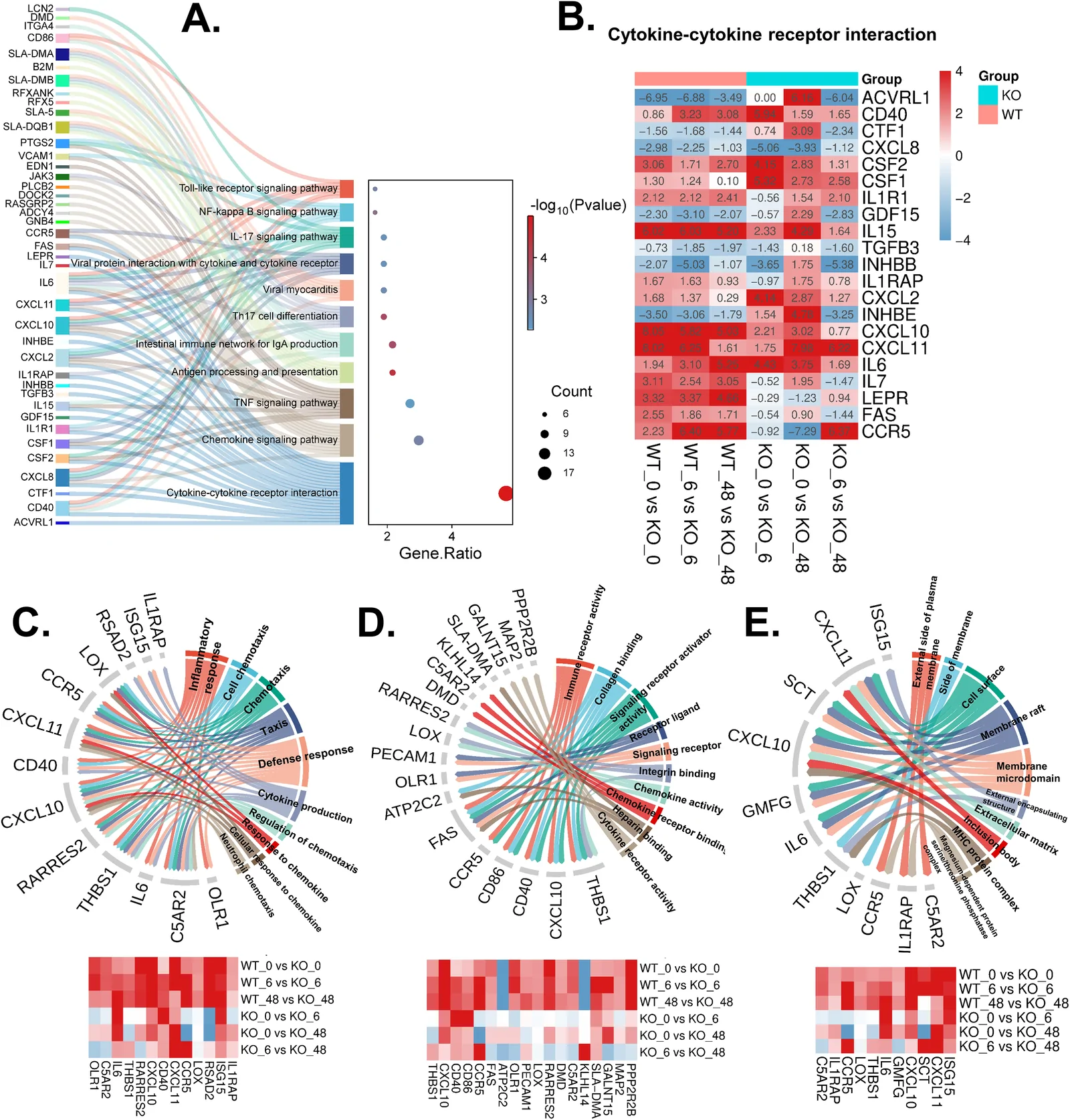
Functional enrichment analysis of conserved shared DEGs between WT and *PD-L1* KO PRRSV-infected iPAM cells at three time points. (**A**) Illustration of immune associated enriched pathways. (**B**) Expression pattern of genes assigned to the top most enriched pathway Cytokine-cytokine receptor interaction. GO-based chord diagram illustrate representative genes and enriched top 10 significant GO terms. (**C**) Biological process. (**D**) Molecular function. (**E**) Cellular component. The heatmaps below each panel represent the relative expression patterns of the genes included in the respective above GO category. WT: Wild type; KO: Knockout.

### WGCNA and functional annotation of significant modules

WGCNA was performed to identify co-expressed gene modules that represent coordinated BP altered by PRRSV infection in *PD-L1* KO cells. A sample clustering tree was constructed to check for outliers and then WGCNA was applied. Next, a setting or condition was applied for WGCNA analysis with a scale-free topology (soft threshold power = 18, scale-free signed r^2^ = 0.8), eigengene clustering with mergeCutHeight = 0.25, and a minimum module size of 30 (S3 Fig). A high spatial clustering dendrogram with dissimilarity was obtained after the dynamic tree cut, and the dynamic tree margin defined multiple co-expression modules (Fig 7A). The module-merging step consolidated closely related modules, resulting in a final set of 8 major modules, shown in blue, dark turquoise, dark green, black, green, grey-yellow, and light yellow colours, along with the grey colour module representing unassigned genes. The WGCNA eigengene analysis showed the expression of each module, and, among all modules, the blue colour module showed a strong positive activation with time (Fig 7B and S3 Fig). A correlation heatmap was generated to illustrate the relationships between modules and traits using Pearson’s correlation coefficient (Fig 7C). Among eight modules, blue colour module showed a strong positive association across essentially all contrasts (Pearson r = 0.971–0.997) (Fig 7C). The findings revealed that the blue colour module, comprising a total of 1763 DEGs, presented the strongest correlation with all traits.

**Fig 7:**
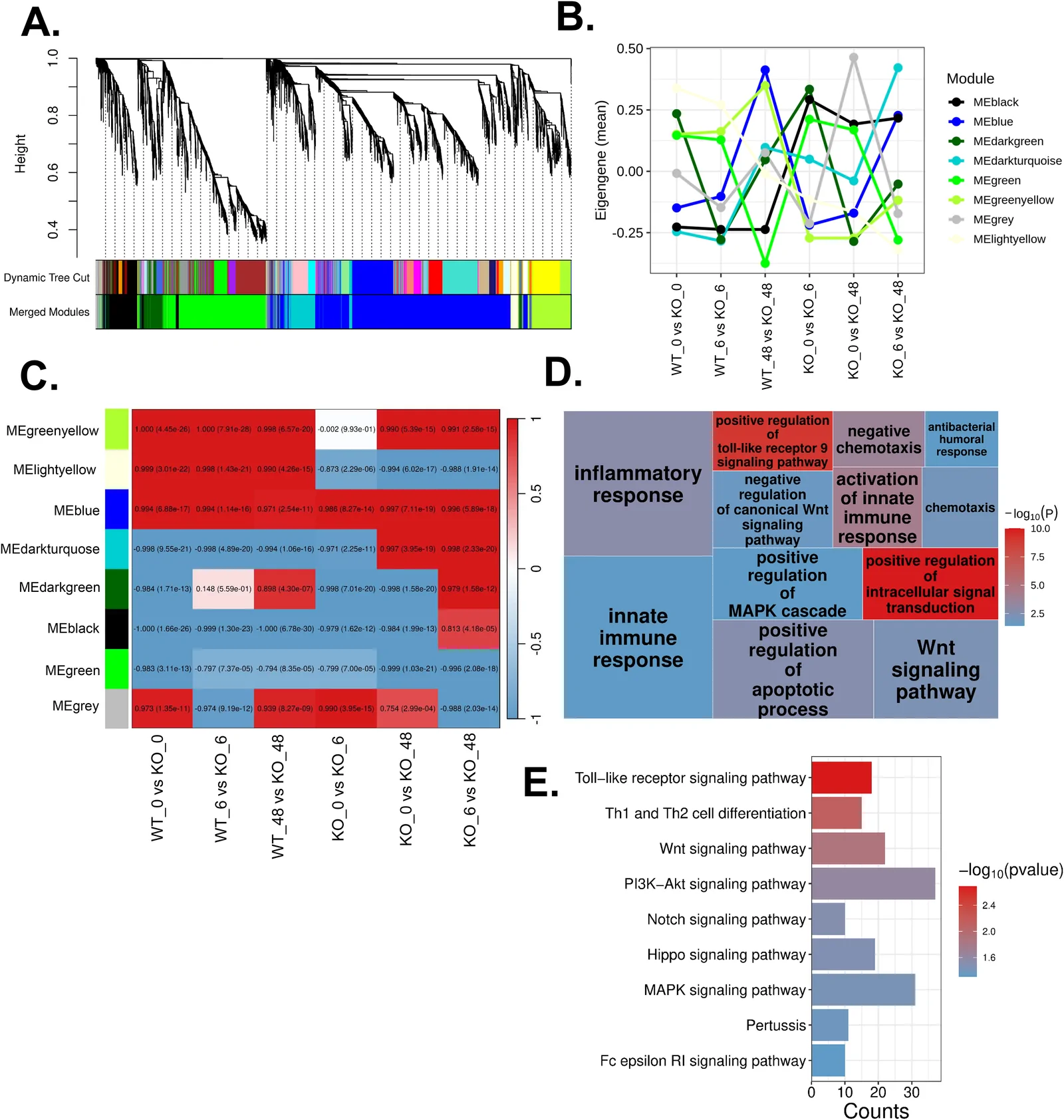
Weighted gene co-expression network analysis. (**A**) Module eigengene expression patterns across the six experimental conditions. Each line represents the mean eigengene value of an individual module. (**B**) Hierarchical clustering dendrogram and module assignment. (**C)** Module and trait relationship correlation heatmap. (**D**) Treemap visualization of the representative biological processes enriched in blue module. (**E**) KEGG pathway enrichment analysis of genes in blue module.

Functional analysis of this module revealed important key GO BP terms, among which positive regulation of intracellular signal transduction was the most significantly (p < 0.05) enriched (Fig 7D). DEGs such as *PDGFRB*, *VWF*, *ERBB2*, *TLR9*, *PTPRJ*, and *MID2* were also enriched in GO BP terms. Other immune-related BP GO terms, including positive regulation of Toll-like receptor 9 signaling pathway, activation of innate immune response, negative chemotaxis, inflammatory response, positive regulation of apoptotic process, Wnt signaling pathway, chemotaxis, negative regulation of canonical Wnt signaling pathway, innate immune response, positive regulation of MAPK cascade, and antibacterial humoral response were also significantly (p < 0.05) enriched in this study. Moreover, KEGG pathway analysis showed that TLR signaling pathway was the most enriched immune pathway (Fig 7E). A full list of KEGG pathways is provided in S6 Table.

### Construction of a PPI network, module analysis, and functional annotation

Subsequently, we constructed a PPI network with DEGs collected from the blue colour module using a STRING database with 1528 nodes and 3894 edges. The analyzed PPI network table is provided in S6 Table. From the entire PPI network, the most relevant module was identified using the MCODE plugin with a node score cut-off of 0.2 and degree cut-off of 2 with k-core  >  6 (Fig 8). Among these 20 nodes, *PXDN* was the hub gene with the highest degree of connectivity. Genes belonging to this module were strongly associated with significantly (p < 0.05) enriched immune allied pathways such as the PI3K-Akt signaling, Toll-like receptor signaling, viral myocarditis, and natural killer cell-mediated cytotoxicity (Fig 8C).

**Fig 8:**
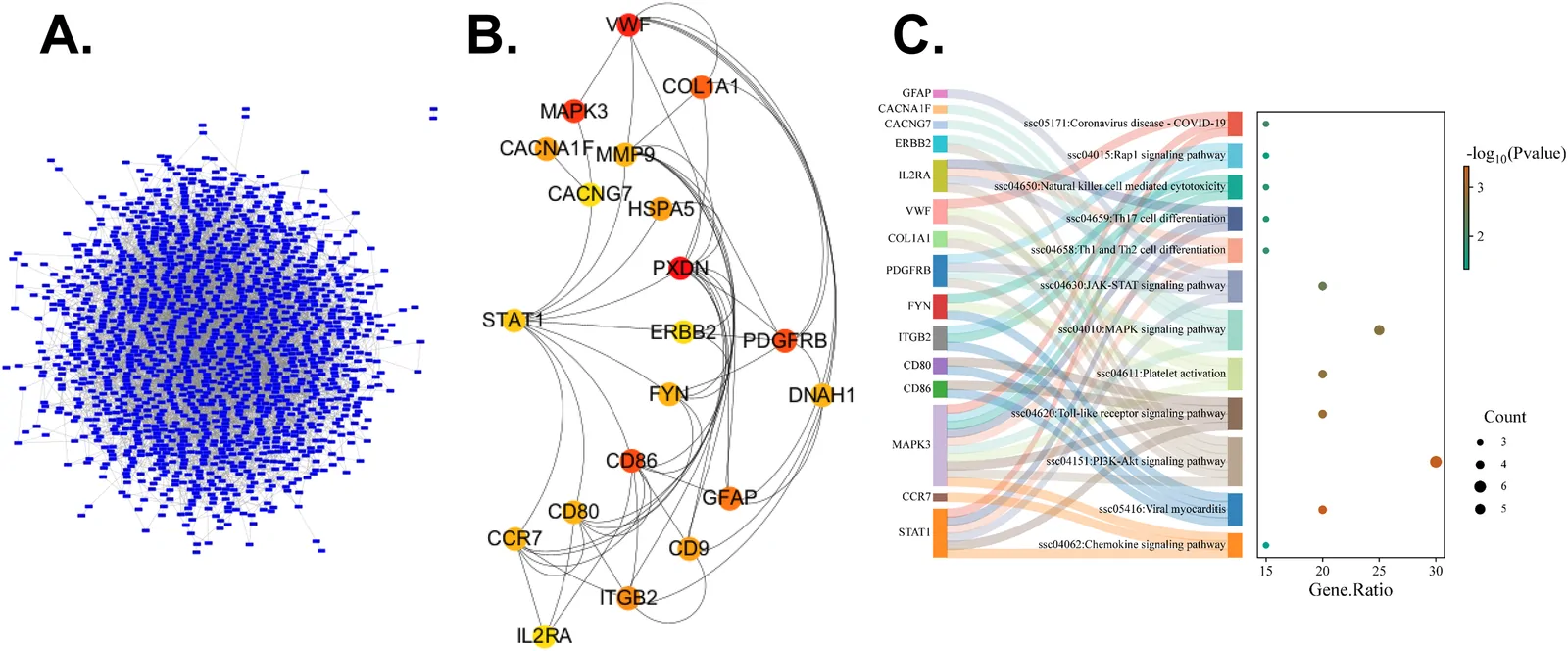
(A) PPI network constructed from genes in the blue module, consisting 1528 nodes and 3894 edges. (B) Top-ranked subnetwork module with 20 nodes extracted from PPI network. (C) KEGG pathway enrichment analysis showing immune-associated pathways significantly enriched among module genes.

## Discussion

PRRSV exhibits a selective tropism for monocyte/macrophage lineage cells, in which it suppresses host innate immune responses to cause chronic infection [3,27]. Among different mechanisms, the PD-1/*PD-L1* axis has emerged as an important immunoregulatory pathway. A previous study suggested that increased *PD-L1* expression in antigen-presenting cells (APCs) is associated with impaired antiviral immunity through its promotion of PRRSV survival and suppression of T cell activation and function during prolonged viral infections [3,6,28]. Consistent with this, earlier studies have reported that suppression of immune checkpoints, including *PD-L1*, can restore antiviral immune responses during chronic viral infections [29,30]. Here, we used CRISPR/Cas9 to KO the *PD-L1* immune checkpoint gene in iPAMs to investigate how its deletion reshapes host transcriptional trajectories during PRRSV infection. We successfully identified a *PD-L1* KO clone #35 and then compared viral replication kinetics in KO cell with WT. Subsequently, we performed a comprehensive RNA-seq analysis at 0, 6, and 48 h post-infection. Interestingly, RT-qPCR analysis confirmed a reduction in viral gene expression in *PD-L1* KO iPAM cells compared to that in WT cells post-PRRSV infection at 48 h. Here, we offer in-depth analysis of the temporal transcriptomic variation in *PD-L1* KO iPAMs during PRRSV infection, allowing a better understand of mechanisms underlying viral persistence and immune evasion.

Time-course RNA-seq profiling at 0, 6, and 48 h post-infection indicated that *PD-L1* KO clone #35 exhibited variation in transcriptional responses during PRRSV infection, with the most noticeable differences observed at 48 h. PCA and correlation analyses showed that transcriptomic patterns varied according to both infection time point and *PD-L1* KO status, collectively accounting for 79% of transcriptomic variance. Moreover, at 48 h post-infection, both WT and KO samples separated from their respective early time-point groups, showing major transcriptional reprogramming at the peak PRRSV replication phase. These findings suggest that *PD-L1* disruption may be associated with altered macrophage transcriptional responses to PRRSV infection; however, clone-specific effects cannot be excluded. Our findings aligns with previous transcriptomic studies showing that PRRSV induces time-dependent remodeling of macrophage responses, with maximal immune dysregulation occurring at 24–72 h post-infection [31]. In our study, the identities of several top-ranked DEGs provided clues regarding the nature of this altered response. Upregulation (13.49-fold at 0 h) of *ADIPOQ*, an anti-inflammatory adipokine, suggests baseline metabolic reprogramming in *PD-L1* KO cells [32]. Similarly, *MGP* and *LRRTM4* (highest at 6 h and 48 h, respectively) are associated with extracellular matrix remodeling and neuronal signaling pathways, respectively [33,34]; *GPR18*, *KCNK17*, and *CTHRC1* upregulation suggests possible activation of G protein-couple receptor signaling, ion channel activity, and collagen remodeling in *PD-L1* KO cells, respectively [35–37]. Collectively, these findings indicate that *PD-L1* KO may be associated with altered antiviral responses and broader cellular remodeling during prolonged PRRSV infection.

Existing literature indicated that PRRSV exploits specific negative regulatory checkpoints within the NF-κB and T-cell exhaustion pathways, effectively silencing pro-inflammatory signals and blunting functional T-cell activation [6,38]. In this study, we examined expression of the associated markers. Among the NF-kB signaling allied genes, SOCS family members (*SOCS1*, *SOCS2*, *SOCS3*, *SOCS4*) and negative regulators of NF-κB signaling (*NFKBIA*, *NFKBID*, *NFKBIZ*) were reported to be highly upregulated during PRRSV infection in PAMs due to their exploitation by viruses [3,39,40]. Furthermore, these studies suggest that the named proteins facilitate PRRSV replication and immune evasion [3,39,40]. Interestingly, our result showed an opposite trend, with these inhibitors and suppressors consistently reduced in KO cells during infection, suggesting sustained NF-kB activation and cytokine signaling. Other critical innate immune effectors, including *ISG15*, *MX1*, and *MX2*, were significantly upregulated in the *PD-L1* KO transcriptome, signaling the establishment of a robust intracellular defense architecture against PRRSV [41–43]. Interferon-stimulated genes such as *MX1*, *MX2*, and *ISG15* are established antiviral effectors, and previous PRRSV studies have shown that *ISG15* can promote an antiviral state in PAMs [44].

In addition to changes in NF‑κB‑related pathways, the reduced expression of several T cell–associated exhaustion markers in *PD‑L1* deficient cells (including *PDCD1*, *LAG3*, *TIGIT*, *PDCD1LG2*, *TOX*, *TOX2*, and *NR4A* family members) points to a possible modulation of inhibitory transcriptional programs typically to chronic antigen exposure and dysfunctional T cell priming. This is because the *PD-L1*/PD-1 pathway is a major regulator of T cell exhaustion during viral infection [6,45]. At the same time, our data showed that other immune checkpoint genes, namely *IDO1* and *HAVCR2* (*TIM-3*), remained elevated, and *CD80* was markedly induced in KO cells. During protracted viral infections, the *HAVCR2* (*TIM-3*) and PD-1 pathways appear to apply non-redundant inhibitory effects, suggesting that they drive CD8^+^ T cell exhaustion through separate molecular cascades [45]. Moreover, blocking the PD-1 pathway does not fully rescue T-cell function, highlighting the contribution of other inhibitory pathways to CD8 + T-cell exhaustion [28,45]. Therefore, these results suggest a possible shift in immune checkpoint–related regulatory programs in *PD-L1* KO cells during PRRSV infection. Collectively, the altered expression of antiviral genes, inhibitory checkpoint markers, and compensatory regulators such as *IDO1* and *HAVCR2* indicates that *PD-L1* disruption may be associated with remodeling of macrophage immune-regulatory transcriptional signatures. However, since the data were obtained from iPAMs and analyzes were based on a single KO#35 clone, it is not possible to directly conclude effects on T-cell exhaustion, and clone-specific transcriptional effect cannot be ruled out.

A major strategy of PRRSV immune evasion is downregulation of MHC molecules to prevent T cell surveillance [6,46,47]. Here, *PD-L1* KO exhibited upregulation in MHC I (*SLA2/5*, *B2M*) and MHC II (*SLA-DMA*, *DMB*, *HLA-DRA*, *DQB1*, *DOB*) genes (> 4-fold at 6 h), co-stimulators (*CD80*, *CD86*, *CD40*, *CD48*), and regulators (*CIITA*, *RFXANK*, *RFX5*). These transcriptional changes suggest that *PD-L1* KO may be associated with enhanced MHC-related transcriptional activity in macrophages, potentially counteracting PRRSV-mediated suppression of antigen presentation [6,46,47]. Although, further functional validation is needed to determine whether this reflects restored antigen presentation capacity.

Our cytokine and chemokine expression profiling revealed that *PD-L1* KO restructured cytokine programs in PRRSV-infected iPAMs toward a more antiviral, less classically pro‑inflammatory state, in a way that both agrees and contrasts with prior PRRSV studies. Sustained upregulation of *CXCL10* and *CXCL11* (> 5-fold) was particularly significant in our results and has been previously reported during PRRSV infection [48,49]. However, *CXCL10* and *CXCL11* are critical *CXCR3* ligands related to monocyte and T cell stimulation and interferon-modulated antiviral responses [49–53]. Moreover, high expression of *ISG15* and *CSF2*, which act as protective antiviral/immune-modulating factors in PRRSV infection, was detected in cells 0–48 h post-infection in the present study [41,54]. Therefore, persistent upregulation of *CXCL10*, CXCL11, *ISG15*, and *CSF2* from early to late time points suggests that *PD-L1* KO reprograms cytokine output toward an antiviral and immune-activating chemokine landscape. In contrast, expression of key pro-inflammatory factors such as *TNF* and *CXCL8* (*IL8*), which are reported to be triggered during PRRSV infection, was reduced in *PD-L1* KO cells [3,16]. Similarly, *PD-L1* KO iPAMs displayed robust and sustained upregulation of multiple ISGs (*IFIT1*, *IFIT1*, *IFIT3*, *IFI44*, *IFI44L*, *IFI6*, and *IFIH1*) from 0 to 48 h, together with a temporary surge of different *IFN‑ALPHA*, *IFN‑OMEGA-6*, and *IFN-DELTA-6* ligands at 6 h. Upregulation in the expression of these ISGs and type I/III Interferons is reported to restrict replication of different viruses, including PRRSV [55]. In addition, expression of *IL-15*, which promotes T and natural killer (NK) cell function when administered as an adjuvant in vaccines against PRRSV, was upregulated at 0, 6, and 48 h [56]. *IL10* and *TNF*, which are crucial for establishing PRRSV infection, promoting immune evasion, and modulating apoptosis in pigs, were downregulated in the present study [57,58]. Taken together, our results show that *PD-L1* deletion in iPAMs may be reshaped the PRRSV-driven cytokine transcriptomic network from a profile characterized by ISG suppression, *IL‑10*- or *TNF*‑dominated dysregulation, and mixed inflammatory chemokine storm to one of a more coordinated antiviral response.

Furthermore, 291 overlapping DEGs were used for functional enrichment analysis, which identified 45 significantly enriched KEGG pathways (p < 0.05). Significant KEGG terms related to indiscriminate immune signaling, including cytokine-cytokine receptor interactions, were found to be the most highly significant enriched pathways from the overlapping DEGs. Cytokine-cytokine receptor interactions showed prominent upregulation of *CXCL10*, *CXCL11*, *IL6*, and *IL15* (> 3-fold), which is consistent with previous transcriptomic studies showing these to be core responders in PRRSV-infected macrophages [59]. GO terms highlighted inflammatory response (BP), immune receptor activity (MF), and external plasma membrane localization (CC) as the top hits, suggesting that transcriptomic changes in the KO cells were mainly related to immune signaling and membrane-associated activation.

WGCNA was implemented to identify core gene modules and hub regulators that organize the immune landscape in *PD-L1* KO iPAMs during PRRSV challenge. The blue colour module displayed a strong positive association with essentially all contrasts and encompassed 1,763 DEGs, indicating that it captured a core transcriptional program tightly linked to infection and *PD-L1* KO. The GO enrichment analysis of genes in the blue colour module showed that positive regulation of intracellular signal transduction was the most highly represented GO BP term. DEGs such as *PDGFRB*, *VWF*, *ERBB2*, *TLR9*, *PTPRJ*, and *MID2* were enriched. Among the DEGs, *PDGFRB* (tyrosine kinase receptor) and *ERBB2* (transmembrane tyrosine kinase receptor), which have been implicated in viral entry, vascular adhesion, oncogenic signaling, and intracellular trafficking in previous studies, were found to be downregulated in *PD-L1* KO cells, suggesting reduced support for virus infection [60–63]. However, genes such as *TLR9*, *PTPRJ*, and *MID2* (E3 ubiquitin ligase), which are involved in antiviral immunity, were downregulated in our KO cells, likely reflecting a broader transcriptional shift in the KO cells [64–66].

In addition, KEGG enrichment analysis of the blue colour module highlighted Toll-like receptor signaling as the top-ranked immune pathway, which plays a critical role in the immune response to PRRSV infection [67]. Notably, enrichment of Toll-like receptor signaling cascade in the blue colour module, driven by upregulated DEGs such as *CD86*, *IFN-ALPHA-8*, *CD80*, *IFN-ALPHA-15*, *IRF5*, and *TLR6*, aligns with our observation of enhancements in antigen presentation and interferon responses in *PD-L1* KO iPAMs. Genes like *TLR6*, *IFN-ALPHA-8*, and *IFN-ALPHA-15* are essential to initiating inflammatory and antiviral signaling through NF-κB-dependent mechanism [68,69]. Similarly, *IRF5* is a key transcription factor that links innate receptor activation to inflammatory and antiviral gene programs [70]. Upregulation of *CD80* and *CD86* complements MHC I/II increases, linking innate activation to improved co-stimulation, which is typically impaired during PRRSV infection [71].

A PPI network was constructed to explore the interaction landscape of genes in the blue colour module. PPI network analysis of DEGs in this module identified a key MCODE module with *PXDN* as the top hub (highest degree) enriched in the PI3K-Akt, Toll-like receptor, viral myocarditis, and NK cell cytotoxicity pathways (p < 0.05). *PXDN* encodes peroxidasin, an extracellular matrix-associated peroxidase that has been widely reported to be a pan-cancer biomarker with elevated expression linked to reduced T cell infiltration in tumors [51]. Beyond cancer biology, *PXDN* is also involved in host defenses, particularly against gram-negative bacteria [72]. Although *PXDN* has not previously been characterized as a canonical hub gene in PRRSV infection, its centrality in the existing network suggests that it may be a new regulator of the host response to PRRSV. Further, KEGG pathway analysis exposed significant (p < 0.05) enrichment of the PI3K-Akt signaling pathway, indicating its possible exploitation by PRRSV to enhance pathogenesis [73]. This aligns with previous reports and highlights PI3K-Akt’s role in regulating macrophage survival, polarization, and inflammatory balance, suggesting that *PD-L1* loss could possibly influences macrophage activation and antiviral responses during PRRSV infection [74].

In conclusion, these findings provide transcriptomic evidence that *PD-L1* deficiency is allied with altered macrophage responses to PRRSV infection in an iPAM model, shifting gene expression toward a more antiviral and immunostimulatory profile. *PD-L1* KO cells showed reduced viral gene expression, sustained induction of interferon-responsive genes and *CXCL10*/*CXCL11*-centered chemokine signaling, enhanced MHC class I/II and co-stimulatory molecule expression, and attenuation of multiple exhaustion-associated checkpoint programs. Although compensatory pathways such as those mediated by *IDO1* and *HAVCR2* remained elevated, the KO cells exhibited lower expression of several immunosuppressive features characteristic of PRRSV infection. Network and pathway analyses revealed that the transcriptional changes observed in *PD-L1* KO cells following PRRSV infection involved coordinated modulation of cytokine signaling, antigen processing and presentation, Toll-like receptor signaling, and other innate immune pathways. WGCNA identified a dominant blue module enriched in different innate immune pathways, and PPI network analysis highlighted *PXDN* as a hub gene within clusters associated with PI3K-Akt signaling and NK cell-mediated cytotoxicity. Overall, our results provide new insight into transcriptional changes in *PD-L1* KO iPAMs during the early and peak phases of PRRSV infection and contribute to a better understanding of the association between *PD-L1* and PRRSV infection mechanisms. However, further comprehensive *in-vitro* and *in-vivo* studies are needed to confirm the physiological relevance and long-term effects of these findings.

Despite its strengths, this study had several limitations. A limitation of this study is that downstream PRRSV infection and transcriptomic analyzes were performed using a single *PD-L1* KO clone, KO#35. So, although the observed changes are consistent with disruption of *PD-L1* expression, clone-specific effects resulting from clonal selection, genetic background variation, or potential off-target events cannot be fully excluded. Future studies using multiple independently generated *PD-L1* KO clones and/or rescue experiments restoring *PD-L1* expression will be required to confirm that the observed phenotypes are specifically attributable to *PD-L1* loss. Additionally, our transcriptomic analysis identified DEGs, hub genes, cytokine-related signatures, and enriched pathways associated with *PD-L1* deficiency during PRRSV infection, these observations were not validated experimentally using qRT-PCR, ELISA, western blotting, or functional assays. In addition, this study was conducted using an in vitro iPAM model, and although *PD-L1* KO reduced viral gene expression, the in vivo relevance of this antiviral effect remains unclear. The highly virulent PRRSV-2 (JBNU-23-N05) strain used in this study appears to exhibit moderate pathogenicity with strong immunomodulatory activity [16], with the most pronounced transcriptional changes observed 48 h post-infection. However, because the analysis was limited to a relatively short infection period, the long-term effects of *PD-L1* deficiency on antiviral defense, immune adaptation, and viral persistence could not be assessed. Nonetheless, the strength of this study lies in the observation that *PD-L1* KO showed reduced viral gene expression in vitro and was associated with broad immune reprogramming toward an antiviral and immunostimulatory state. Future studies should validate the functions of key DEGs, hub genes, and pathways at the protein and mechanistic levels; investigate *PD-L1* interactions with innate immune signaling and compensatory checkpoint pathways; and extend these findings by applying prolonged infection models and in vivo validation. WGCNA was performed using 18 samples distributed across six experimental groups, the inferred coexpression modules should be regarded as exploratory. The relatively small number of biological replicates may influence gene-gene correlations and module assignments, and formal module-preservation or resampling analyses were not performed. Therefore, validation of the identified modules in larger independent datasets will be required to establish their reproducibility.

## Supporting information

S1 Fig (A) List of primers used in this study. (**B**) Cleavage assay for evaluation of CRISPR/Cas9-mediated target site disruption. WT: Wild type; KO: Knockout.
(PPTX)

S2 Fig Differential expression profiles between WT and *PD-L1* KO iPAM cells across PRRSV infection time points. (**A**) Volcano plots shows differential expression profiles between WT and *PD-L1* knockout iPAM cells across PRRSV infection time points. Significance was determined with p_adj_ ≤ 0.05 and log_2_ fold-change ≥ 1.5. (**B**) Hierarchical clustering and heatmap of the Top 500 DEGs in WT and *PD-L1* KO iPAM cells across PRRSV infection time points using cut-off criteria of p_adj_ ≤ 0.05 and log_2_ fold-change ≥1.5. WT: Wild type; KO: Knockout.
(PPTX)

S3 Fig (A) Analysis of network topology for selection of the soft-thresholding power in weighted gene co-expression network analysis. (**B**) Boxplots of module eigengene expression patterns across the six experimental conditions for the identified co-expression modules (black, blue, darkgreen, darkturquoise, green, greenyellow, grey, and lightyellow). Expression values are presented as z score–normalized module expression levels.
(PPTX)

S1 Table Summary of RNA-seq sequencing quality, read alignment, and gene-assignment statistics for individual samples. (XLSX)

S2 Table Total differentially expressed genes (DEGs) at cut-off of padj < 0.05 and log2 fold change ≥ 1.5. (XLSX)

S3 Table Overlapping DEGs between six contrasts. (XLSX)

S4 Table Expression profile of checkpoint- and exhaustion-associated genes, NF-kB signaling pathway allied genes, MHC class I and II allied genes MHC class co-stimulation genes, cytokines and their receptors during PRRSV infection. (XLSX)

S5 Table GO and KEGG pathway enrichment analyses of overlapped DEGs from all six comparison WT_0 vs KO_0|WT_6 vs KO_6|KO_48 vs KO_48|KO_0 vs KO_6|KO_0 vs KO_48|KO_6 vs KO_48. (XLSX)

S6 Table List of DEGs within the WGCNA blue module, along with their associated GO terms, KEGG pathways, and PPI network analysis. (XLSX)

S1 Raw Images Original uncropped western blot images underlying Fig 1D. Full-uncropped images of PD-L1 and GAPDH immunoblots from WT and PD-L1 KO clone #35 iPAM cells cultured with or without IFN-γ stimulation (100 ng/mL for 24 h). PD-L1 was detected at approximately 45 kDa, and GAPDH was detected at approximately 36 kDa. Images were acquired using a Bio-Rad GelDoc XR+ imaging system. Lane identities and IFN-γ treatment conditions are indicated above and below each blot, respectively. PD-L1 and GAPDH were detected on separate membranes using protein lysates from the same experimental samples.
(PDF)
