# Supplementary material for: CRISPR/Cas9-mediated PD-L1 knockout reshapes temporal transcriptomic responses to PRRSV infection in porcine alveolar macrophages
Source: PLoS One. 2026 Nov 10;21(11):e0355142. doi: 10.1371/journal.pone.0355142 (PMC13750538; doi:10.1371/journal.pone.0355142)
Supplement: Supplementary file 1 [file pone.0355142.s010.pdf]

S1_raw_images

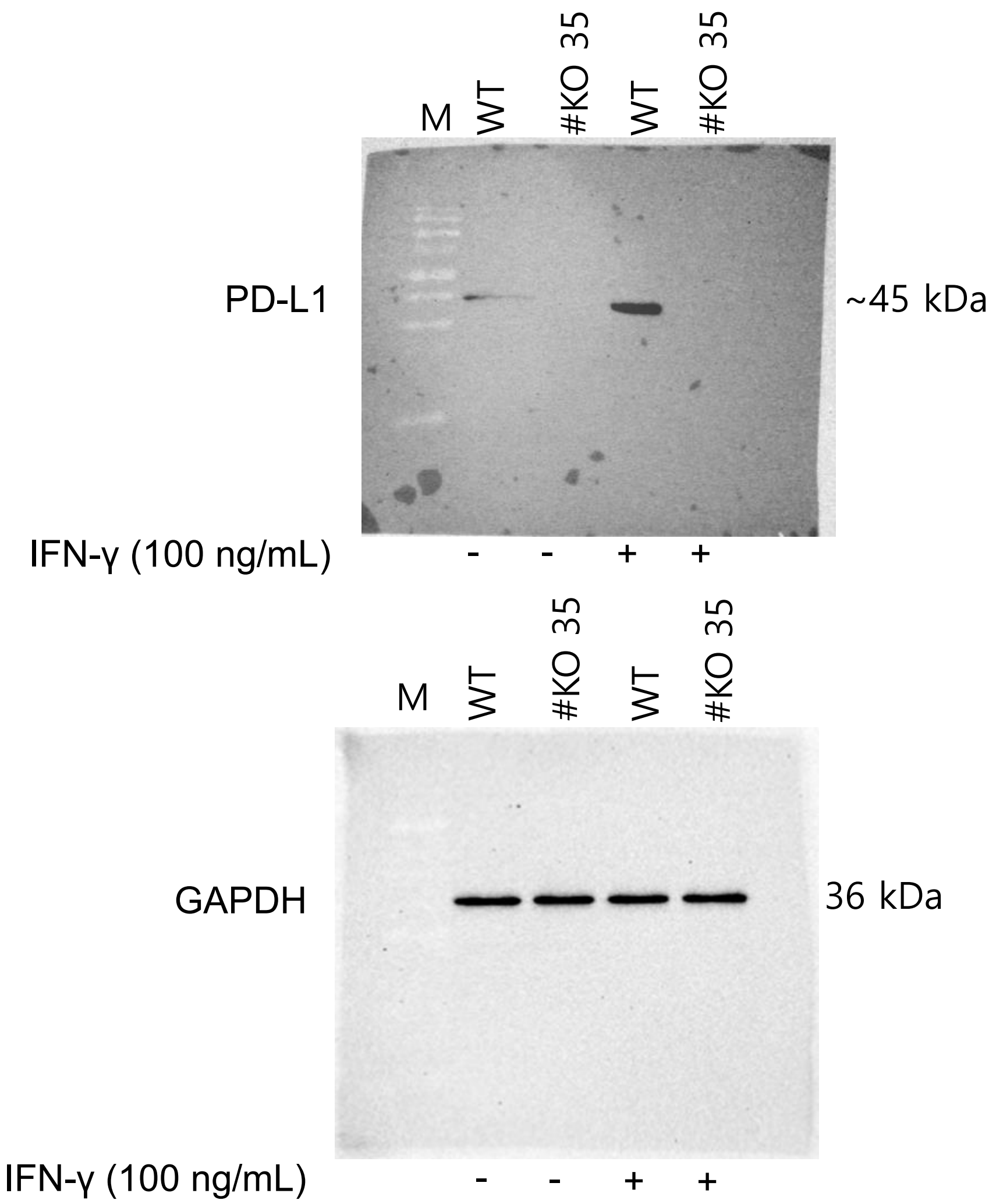

S1 Raw images. Original uncropped western blot images underlying Fig 1D. Full-uncropped images of PD-L1 and GAPDH immunoblots from WT and PD-L1 KO clone #35 iPAM cells cultured with or without IFN-γ stimulation (100 ng/mL for 24 h). PD-L1 was detected at approximately 45 kDa, and GAPDH was detected at approximately 36 kDa. Images were acquired using a Bio-Rad GelDoc XR+ imaging system. Lane identities and IFN-γ treatment conditions are indicated above and below each blot, respectively. *PD-L1* and GAPDH were detected on separate membranes using protein lysates from the same experimental samples.
